# A preliminary study of the chemical composition and bioactivity of *Bombax ceiba* L. flower and its potential mechanism in treating type 2 diabetes mellitus using ultra-performance liquid chromatography quadrupole-time-flight mass spectrometry and network pharmacology analysis

**DOI:** 10.3389/fnut.2022.1018733

**Published:** 2022-10-13

**Authors:** Kehong Yin, Jinmei Yang, Fang Wang, Zhenxing Wang, Ping Xiang, Xing Xie, Jian Sun, Xuemei He, Xuechun Zhang

**Affiliations:** ^1^Key Laboratory for Forest Resources Conservation and Utilization in the Southwest Mountains of China, Ministry of Education, College of Life Science, Southwest Forestry University, Kunming, China; ^2^Institute of Environmental Remediation and Human Health, Southwest Forestry University, Kunming, China; ^3^National R&D Center for Freshwater Fish Processing, College of Health, Jiangxi Normal University, Nanchang, China; ^4^Guangxi Key Laboratory of Fruits and Vegetables Storage-Processing Technology, Guangxi Academy of Agricultural Sciences, Nanning, China

**Keywords:** *Bombax ceiba* L. flower, phytochemical composition, biological activity, UPLC/Q-TOF-MS, network pharmacology

## Abstract

This study aimed to preliminary investigate the phytochemistry, bioactivity, hypoglycemic potential, and mechanism of action of *Bombax ceiba* L. flower (BCF), a wild edible and food plant in China. By using methanol extraction and liquid-liquid extraction, the crude extract (CE) of BCF and its petroleum ether (PE), dichloromethane (DCM), ethyl acetate (EtOAc), n-butanol (n-BuOH), and aqueous (AQ) fractions were obtained, and their chemical components and biological activities were evaluated. Further high-performance liquid chromatography (HPLC) analysis was carried out to identify and quantify the active constituents of BFC and its five fractions, and the phytochemical composition of the best-performing fraction was then analyzed by ultra-performance liquid chromatography quadrupole-time-flight mass spectrometry (UPLC/Q-TOF-MS). Finally, a network pharmacology strategy based on the chemical profile of this fraction was applied to speculate its main hypoglycemic mechanism. Results revealed the excellent biological activities of BCF, especially the EtOAc fraction. In addition to the highest total flavonoid content (TFC) (367.72 μg RE/mg E) and total phenolics content (TPC) (47.97 μg GAE/mg E), EtOAc showed the strongest DPPH⋅ scavenging ability (IC_50_ value = 29.56 μg/mL), ABTS⋅^+^ scavenging ability (IC_50_ value = 84.60 μg/mL), and ferric reducing antioxidant power (FRAP) (889.62 μg FeSO_4_/mg E), which were stronger than the positive control BHT. EtOAc also exhibited the second-best α-glucosidase inhibitory capacity and second-best acetylcholinesterase (AChE) inhibitory capacity with the IC_50_ values of 2.85 and 3.27 mg/mL, respectively. Also, EtOAc inhibited HepG2, MCF-7, Raw264.7, and A549 cell with IC_50_ values of 1.08, 1.62, 0.77, and 0.87 mg/mL, which were the second or third strongest in all fractions. Additionally, HPLC analysis revealed significant differences in the compounds’ abundance between different fractions. Among them, EtOAc had the most detected compounds and the highest content. According to the results of UPLC/Q-TOF-MS, 38 compounds were identified in EtOAc, including 24 phenolic acids and 6 flavonoids. Network pharmacological analysis further confirmed 41 potential targets of EtOAc in the treatment of type 2 diabetes, and intracellular receptor signaling pathways, unsaturated fatty acid, and DNA transcription pathways were the most possible mechanisms. These findings suggested that BCF was worthwhile to be developed as an antioxidant and anti-diabetic food/drug.

## Introduction

*Bombax ceiba* L. (BC) is a *Bombax* plant belonging to the *Bombacaceae* family, which is mainly distributed in Asia, Africa, and Oceania (1). As an ethnomedicine plant, BC is reported to be rich in active components, such as polysaccharides, phenolics, flavonoids, and neolignans, and is often used to treat diarrhea, fever, hepatitis, impotence, etc. (2). Many studies on BC have indicated its various bioactive potentials, such as the C-flavonol glucoside and triterpenoid compounds, and the bioactive compounds in BC leaves and barks, have been reported to have good hypoglycemic and hypolipidemic effects (3). Moreover, the antiangiogenic activity (4), anti-Hepatitis B virus activity (5), sexual function-enhancing activity (6), and hypotensive activity (7) were also reported for the compounds or extracts from the various parts of BC, including stem barks, roots, stem barks. However, relatively few studies have been reported for the flower of BC (BCF), in its main edible part.

As reported, BCF exhibited excellent antioxidant activities including 1,1-diphenyl-2-picrylhydrazyl (DPPH) radical-scavenging activity, oxygen radical absorbance capacity (ORAC), reducing power, and inhibition of phosphatidylcholine liposome peroxidation (8). In addition, it also showed promising abilities to scavenge hydroxyl free radicals, against lipid peroxidation induced by ascorbyl radicals and peroxynitrite, and inhibition ability on myeloperoxidase (9). Apart from the above, Abd-Elhaki et al. (10) reported the preventive and therapeutic effects on ethanol gastric injury of BCF. Wanjari et al. (11) found that BCF could prevent CCl_4_-induced hepatotoxicity. Further, it also demonstrated potent *in vitro* anticancer and antidiabetic activities (12, 13). Therefore, BCF is considered an excellent naturally occurring antioxidant, displaying a variety of biological activities, and is worth further exploring.

Currently, many dietary sources, such as plants, herbs, spices, and herbal extracts, have been recognized as good sources of natural antioxidants (14). It can be useful in the prevention and/or management of oxidative stress-related disorders, including diabetes, cancer, inflammation, thrombosis, and chronic, and neurodegenerative diseases (15–17). When compared with synthetic antioxidants, natural antioxidants have the advantage of being non-toxic and having fewer side effects (18–20). For example, *dendrobium* polyphenols, bergamot polyphenolic, gallic acid, and other natural antioxidants can exert direct antidiabetic effects by protecting β-cells, increasing insulin secretion, and consequently improving glucose metabolism (21). Especially, the flavonoid and phenolic acid compounds derived from plants have the advantages of wide sources and easy collection, thus have strong development prospects and are the current research hotspots. Therefore, it is of considerable interest to explore BCF for its antioxidant and other biological activities.

In this study, the crude extract of BCF and its different polarity solvent fractions were obtained by using methanol extraction and liquid-liquid extraction, and their total phenolic and flavonoid contents, antioxidant and anticancer activities, α-glucosidase and acetylcholinesterase (AChE) inhibitory capacities were evaluated to assess their antioxidant, hypoglycemic, and treat Alzheimer’s disease potential. To elucidate the main material basis for BCF to manage type 2 diabetes mellitus (T2DM), the identification and quantification of their active constituents were carried out using high-performance liquid chromatography (HPLC), and the compound identification of the best-performing fraction was performed by ultra-performance liquid chromatography quadrupole-time-flight mass spectrometry (UPLC/Q-TOF-MS). Finally, network pharmacology was used to establish a compound-target-disease network for exploring the potential pharmacological targets and mechanisms of BCF in T2DM treatment. Our study may give new insight into the therapy of T2DM, and provide some valuable references for the further development and utilization of BCF in food/drug processing.

## Materials and methods

### Chemicals and reagents

The 2, 2-diphenyl-1-picrylhydrazyl (DPPH), 2, 2′-azino-bis-(3-ethylbenzothiazoline-6-sulfonic acid) (ABTS), 2, 4, 6-Tris(2-pyridyl)-s-triazine (TPTZ), 2, 6-di-tert-butyl-4-methylphenol (BHT), vitamin C (Vc), were obtained from Solarbio Life sciences (Beijing, China). All chemical reagents including methanol, ethyl acetate (EtOAc), petroleum ether (PE), Dichloromethane (DCM), and n-butanol (n-BuOH) were purchased from Sinopharm Chemical Reagent Co., Ltd. (Shanghai, China). Chromatographic acetonitrile was purchased from Merck (Darmstadt, Germany). Folin-Ciocalteu, pyrogallol, and other analytical grade chemicals were purchased from Aladdin (Shanghai, China). Myricetin, dihydromyricetin, quercetin, resveratrol, apigenin, baicalein, hesperitin, gallic acid, rutin, luteolin, epicatechin, epicatechin gallate, and other standards for HPLC were purchased from Yuanye Bio-Technology (Shanghai, China). Cellulase (3, 000 μ/g) and pectinase (40, 000 μ/g) were purchased from Xiya reagent (Shandong, China). α-Glucosidase (G5003) and acetylcholinesterase (AChE, C3389) were purchased from Sigma-Aldrich (St. Louis, USA). p-Nitrophenol nitrophenol α-D-glycopyranoside (pNPG), acarbose, galantamine, and cisplatin were purchased from Sigma-Aldrich (St. Louis, MO, USA). The HepG2, MCF-7, Raw264.7, and A549 cells were purchased from Conservation genetics CAS Kunming cell bank.

### Sample preparation

BCF was purchased in Yulin city, Guangxi, China. The sun-dried BCF was pulverized and passed through a 60-mesh sieve to obtain powder. 500 g of BCF powder was mixed with distilled water at a ratio of 1:6 (m/v), and the pH was adjusted to 5 with hydrochloric acid (2 M HCl), then 0.04% cellulose (m/m) and 0.01% pectinase (m/m) were added, followed by ultrasound-assisted enzymatic dissociation at 800 W, 50°C for 1 h, the samples were then added with 7 L of methanol to a final concentration of 70% methanol (v/v). Next, ultrasound-assisted extraction was performed also at 800 W, 50°C for 1 h. The supernatant was got by centrifugation, and the residues were repeatedly extracted for one time. All the supernatants were collected and evaporated under reduced pressure at 50°C to obtain crude extract (CE). The CE was dissolved in 1.5 L H_2_O and sequentially enriched twice with an equal volume of DCM (polarity 3.4), PE (polarity 0.1), EtOAc (polarity 4.4), and n-BuOH (polarity 3.7). The different fractions and residual aqueous fraction (AQ) were collected and removed the solvents were under reduced pressure, then lyophilized to get samples. All samples were kept at –20°C for further analysis.

### Determination of total phenolic and flavonoid contents

The total phenolic content (TPC) was determined by the Folin-Ciocalteu method (22). 50 μL of properly diluted samples were mixed with 25 μL of 0.5 M Folin-Ciocalteu reagents in a 96-well microplate and reacted for 5 min at 25°C. A total of 200 μL of Na_2_CO_3_ (7.5%, w/v) was then added and incubated for 30 min at 25°C. Finally, the absorbance was measured at 765 nm by a microplate reader (Biotek, VT, USA). Ethanol was used as blank control. Gallic acid (10–100 μg/mL) was used as the standard, and the results were expressed as μg of gallic acid equivalents per mg of extract (μg GAE/mg E).

The total flavonoid content (TFC) was evaluated using a previous method (23). 50 μL of appropriately diluted sample was mixed with 25 μL of 3% NaNO_2_ solution in a 96-well microplate. After incubation for 6 min, 25 μL of 6% Al (NO_3_)_3_ was added and reacted for another 6 min, then 175 μL of 4% NaOH and 75 μL of 70% methanol were added. The mixtures were incubated at room temperature for 15 min and the absorbance was determined at 510 nm. Ethanol was used as blank control, and rutin (10–100 μg/mL) was used as standard. The results were expressed as μg of rutin equivalents mg extract (μg RE/mg E).

### Determination of antioxidant activity

#### Diphenyl-2-picrylhydrazyl radical scavenging activity

The diphenyl-2-picrylhydrazyl radical (DPPH⋅) scavenging activity was assessed according to the reported method (24). Briefly, 100 μL properly diluted sample solution was mixed with 100 μL of DPPH solution (0.15 mmol/L) in a 96-well plate. After reacting at room temperature in a dark environment for 30 min, the absorbance value (*A*s) was detected at 517 nm. Methanol was replaced with the DPPH solution as a blank control (*A*b), methanol was replaced with the sample as absorbance control (*A*c), and Vc and BHT were used as positive controls. The inhibition rate was calculated using the following formula (1), and the results were expressed as IC_50_ value (μg/mL), which represented the sample concentration to scavenge 50% free radical.


(1) Scavengingrate(%)=(1-(As-Ab)/Ac)×100%

#### Azino-bis-(3-ethylbenzothiazoline-6-sulfonic acid) radical scavenging activity

The azino-bis-(3-ethylbenzothiazoline-6-sulfonic acid) radical (ABTS⋅^+^) scavenging activity was measured according to a slightly modified method (25). Before the assay, the ABTS solution (7 mM) was mixed with potassium sulfate (2.45 mM) and incubated for 12 h at room temperature in a dark environment, and was then diluted with methanol to an absorbance of 0.70 ± 0.02 at 734 nm. For the assay, 50 μL of sample solution was mixed with 200 μL of ABTS working solution in a 96-well plate and reacted for 5 min at room temperature, then the absorbance of samples at 734 nm was measured (*A*s). Equivalently, methanol was replaced with the ABTS working solution as a blank control (*A*b), methanol was replaced with the sample as absorbance control (*A*c), Vc and BHT were used as positive controls. The scavenging rates were calculated according to equation (1), and the results were expressed as IC_50_ value (μg/mL).

#### Ferric reducing antioxidant power assay

The ferric reducing antioxidant power (FRAP) was determined with the previous method (26). 20 μL of sample solution was mixed with 300 μL of FRAP working solution in a 96-well plate, and protected from light at 37°C. After incubation for 10 min, the absorbance of samples at 593 nm was measured. Methanol was used as a negative control, Vc and BHT were used as positive controls. Ferrous sulfate (10–100 μg/mL) was taken as a standard for the preparation of the standard curve, and the FRAP value was expressed as mg of FeSO_4_ per gram of extract (μg FeSO_4_/mg E).

### Determination of α-glucosidase inhibitory ability

The α-glucosidase inhibitory was evaluated with the method described by Ru et al. (27). A total of 50 μL of the sample with suitable concentration was mixed with 20 μL of 0.1 U/mL α-glucosidase in a 96-well microplate and incubated at 37°C for 10 min, then 50 μL of 5 mM pNPG solution was added and incubated in darkness for 15 min at 37°C. Finally, the reaction was stopped with the addition of 100 μl of 0.2 M Na_2_CO_3_, and the absorbance at 405 nm was recorded (*A*s). Acarbose was used as a positive control, and phosphate buffered saline instead of α-Glucosidase was used as the blank control (*A*b), phosphate-buffered saline instead of the sample was used as the absorbance control (*A*c). The results were expressed as IC_50_ value (mg/mL). The α-glucosidase inhibition rate was calculated as follows:


(2) Inhibitionrate(%)=(1-(As-Ab)/Ac)×100%

### Determination of acetylcholinesterase inhibitory ability

The AChE inhibitory properties with the method described by Kitazawa et al. (28). A total of 50 μL of the sample with suitable concentration was mixed with 90 μL of Ellman’s solution (containing 15 μL of 15 mM ATCI and 75 μL of 3 mM DTNB) in a 96-well microplate and incubated in darkness for 10 min at 30°C. Then 20 μL of 0.2 U/mL AChE was added and kept in darkness for 5 min. Finally, the absorbance at 734 nm was measured (*A*s). Galanthamine was used as positive control, and phosphate buffer in place of acetylcholine was used as the blank control (*A*b), phosphate buffer in place of the sample was used as the absorbance control (*A*c). The results were expressed as IC_50_ value (mg/mL). The inhibition rate was calculated similarly to equation (2).

### Determination of cell proliferation inhibitory ability

The inhibition activities of samples on HepG2, MCF-7, Raw264.7, and A549 cell proliferation were measured using the CCK-8 assay (29). Briefly, cells were seeded in 96-well plates (5 × 10^3^ cells per well) and placed in a cell culture incubator at 37°C. After 24 h of incubation, 10 μL of the suitable concentration of the sample was added and incubated for 24 h, followed by the addition of CCK-8 solution (10 μL), cells were incubated for another 4 h, then the absorbance was measured at 450 nm. Cisplatin was used as a positive control. The cell proliferation inhibitory ability was expressed as the IC_50_ value (μg/mL).

### High-performance liquid chromatography analysis

HPLC analysis was performed by an HPLC 1260 (Agilent Technologies, CA, USA) equipped with a diode array detector (DAD) (30). Chromatographic separations were performed on the C18 liquid chromatography column (250 × 4.6 mm, 5 μm, Greenherbs Science and Technology) at 25°C, distilled water containing 0.1% formic acid (A) and acetonitrile (B) was used as the mobile phases with a flow rate of 0.8 mL/min. The gradient elution condition was as follows: 0–12 min, 2–8% B; 12–15 min, 8–13% B; 15–30 min, 13–18% B; 30–50 min, 18–30% B; 50–60 min, 30–50% B; 60–70 min, 50–70% B; 70–80 min, 70–90% B; 80–85 min, 90–100% B; 85–90 min, 100–2% B. Samples were filtered through nylon syringe filters (0.22 μm) and injected with a volume of 20 μL. The chromatogram was detected from 200 to 400 nm, and the peak areas were recorded. Finally, compounds were identified and quantified by comparing the retention times with standards. In this study, standards curves were prepared using gallic acid (*y* = 29.703x–84.271, *R*^2^ = 0.9999) at 280 nm, myricetin (*y* = 15.74x–65.951, *R*^2^ = 0.9999) at 360 nm, dihydromyricetin (*y* = 25.835x + 50.339, *R*^2^ = 0.9998) at 280 nm, quercetin (*y* = 15.904x–10.892, *R*^2^ = 0.9907) at 360 nm, resveratrol (*y* = 21.538x + 15.061, *R*^2^ = 0.9995) at 280 nm, apigenin (*y* = 21.326x–41.932, *R*^2^ = 0.9987) at 360 nm, baicalein (*y* = 65.901x–458.59, *R*^2^ = 0.9953) at 280 nm, hesperitin (*y* = 46.714x–76.478, *R*^2^ = 0.9995) at 280 nm, rutin (*y* = 15.957x + 3.3269, *R*^2^ = 0.9998) at 256 nm, epicatechin (*y* = 13.438x x.43picate^2^ = 0.9990) at 280 nm, (–)-epicatechin gallate (ECG) (*y* = 14.492x x.49techin ^2^ = 0.9982) at 280 nm, luteolin (*y* = 25.599x x.59uteoli^2^ = 0.9978) at 360 nm, and the concentration of all standards ranged from 20 to 100 μg/mL. The results were expressed as μg of standards per mg of extract.

### Ultra-performance liquid chromatography quadrupole-time-flight mass spectrometry analysis

The UPLC/Q-TOF-MS analysis was performed using a Triple-TOF 5600^+^ quadrupole time-of-flight mass spectrometer from AB-Sciex (31). The liquid phase part used a Shimadzu LC-30 ultra-high performance liquid chromatography system equipped with a Shimadzu SPD-M20A diode array detector. The chromatographic column was a Shim-pack GIST C18 reversed-phase column with a column volume of 75 mm × 2.1 mm, a particle size of 2.0 μm, and a column temperature of 25°C. The elution flow rate was 0.2 mL/min, and the maximum pressure was 100 MPa. The mobile phase was consisted of 0.1% aqueous formic acid (A) and acetonitrile (B), and the optimal elution conditions were: 0–10 min, 95% A and 5% B; 10–27 min, 90% A and 10% B; 27–40 min, 82% A and 17% B; 40–50 min, 70% A and 30% B; 50–55 min, 100% B. Finally, the column was eluted with 95% A and 5% B. The sample concentration was 1.0 mg/mL, and the injection volume was 10 μL, which was filtered through a 0.22 μm nylon membrane before injection. The mass spectrometry parameters were: Scanning mode is electrospray ionization (ESI) source negative ion mode; air curtain gas 40 psi; temperature 550°C; ion spray voltage—4,500 V; ion source atomizing gas 1 and auxiliary gas 2 are both 50 psi; The scanning mass range (m/z) was from 50 to 1500. Finally, the mass spectrometry data were processed with Peak View software, by comparing the precursor ion (m/z, [m-N]-), production (m/z), chromatographic peak retention time (Rt, min), and secondary fragment MS^2^ with reference and database to analyze samples.

### Network pharmacology analysis

The network pharmacology analysis was based on the results of the identification of the compounds by UPLC/Q-TOF-MS, and the information about these compounds and the compound-related targets was collected from the Traditional Chinese Medicine Systems Pharmacology Database (TCMSP),^1^ PubChem database^2^ and sea database^3^ (31, 32). By using “diabetes” as the search term, the names of diabetes target genes were obtained on the GeneCard database,^4^ then the VENNY2.1 software was used to make an intersection pie chart to obtain the common target gene information of diabetes and sample. The common genes were imported into the STRING database^5^ to construct the protein interaction (PPI) network, the species was “Homo sapiens,” and the minimum interaction threshold was medium and “Highest confidence > 0.9.” Next, Gene Ontology (GO) function enrichment analyses were performed using the DAVID 6.8 database.^6^ The target signaling pathway was analyzed by the Kyoto encyclopedia of genes and genome (KEGG) pathway enrichment, with *P* < 0.05 as the threshold, and the count value was sorted. The top 20 signaling pathways were selected and Omicshare^7^ was used to make a visual bubble chart.

### Statistical analysis

All experiments were repeated three times, and the results were shown as mean ± standard deviation. The correlation analysis, significant difference, and principal component analysis (PCA) were performed using R (ver 4.0.3). Origin 2018 and R were used to plot graphs.

## Results and discussion

### Total phenolics and flavonoids content

As shown in Table 1, EtOAc fraction showed the highest TPC (47.97 ± 0.51 μg GAE/mg E), followed by CE, AQ, n-BuOH, DCM, and PE fractions with the contents of 41.67 ± 1.02, 40.63 ± 0.45, 39.82 ± 0.62, 37.69 ± 0.75, and 15.41 ± 0.67 μg GAE/mg E, respectively. Among them, EtOAc was significantly higher than other fractions, while PE was the lowest (*p* < 0.05). Similarly, the TFC of samples in decreasing order were EtOAc, n-BuOH, CE, AQ, DCM, and PE, with the values of 367.72 ± 14.26, 104.31 ± 3.80, 43.54 ± 2.61, 30.87 ± 2.66, 16.88 ± 0.77, and 13.55 ± 2.45 RE/mg E. The highest was still EtOAc, and PE remained the lowest. There was a clear gap between EtOAc and other fractions in TFC, where EtOAc was 8.45 times CE, and 27.14 times PE. The results showed that EtOAc had the best enrichment effect on polyphenols and flavonoids. While previous studies have reported the ethanol extract from BCF had high TPC and TFC, they had no comprehensive comparison of the extraction effects of different solvents, and the standard equivalent substances were different (12, 13).

**TABLE 1:** The total phenolic and flavonoid contents of BCF crude extract and its fractions.

Samples	TPC	TFC
CE	41.67 ± 1.02^b^	43.54 ± 2.61^c^
PE	15.41 ± 0.67^e^	13.55 ± 2.45^e^
DCM	37.69 ± 0.75^d^	16.89 ± 0.77^de^
EtOAc	47.97 ± 0.51^a^	367.72 ± 14.26^a^
n-BuOH	39.82 ± 0.62^c^	104.31 ± 3.80^b^
AQ	40.63 ± 0.45^bc^	30.87 ± 2.66^cd^

TPC, total content (μg GAE/mg E); TFC, total flavonoid content (μg RE/mg E); CE, crude extract; PE, Petroleum ether fraction; DCM, Dichloromethane fraction; EtOAc, ethyl acetate fraction; n-BuOH, n-Butanol fraction; AQ, aqueous phase residue. The columns with different letters represent a significant difference at *p* < 0.05.

### Antioxidant properties

**FIGURE 1:**
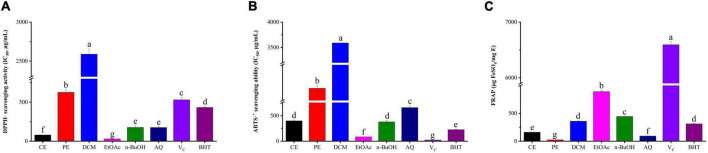
The antioxidant activity of BCF crude extract and its fractions. **(A)** DPPH⋅ scavenging assay, **(B)** ABTS⋅^+^ scavenging assay, **(C)** ferric reducing antioxidant power (FRAP), CE, crude extract; PE, Petroleum ether fraction; DCM, Dichloromethane fraction; EtOAc, ethyl acetate fraction; n-BuOH, n-Butanol fraction; AQ, aqueous phase residue. The columns with different letters represent a significant difference at *p* < 0.05.

DPPH is a stable free radical that could react with the antioxidant, and the fading degree of DPPH solution reflects the scavenging ability of antioxidant compounds, while the ABTS assay involves the transfer of hydrogen electrons and its principle is that oxidants convert ABTS to green ABTS⋅^+^ (33). The DPPH radical scavenging activity of BCF and its fractions were displayed in Figure 1A. Among them, the EtOAc fraction (IC_50_ value = 29.56 ± 1.81 μg/mL) exhibited the strongest DPPH radical scavenging activity, followed by CE, AQ, and n-BuOH fractions with IC_50_ values of 79.46 ± 1.82, 174.98 ± 10.23, and 176.75 ± 23.70 μg/mL. When compared with the positive control Vc (IC_50_ value = 530.24 ± 31.21μg/mL) and BHT (IC_50_ value = 432.12 ± 12.08 μg/mL), it was surprising to find that nearly all fractions were superior to positive control except for PE and DCM (*p* < 0.05). In particular, EtOAc and CE were significantly stronger than other fractions (*p* < 0.05). As displayed in Figure 1B, similarly to the DPPH radical scavenging activity, the EtOAc fraction had the strongest scavenging effect on ABTS free radicals with the IC_50_ value of 84.60 ± 1.56 μg/mL, which was slightly weaker than that of Vc (IC_50_ value = 21.62 ± 1.04 μg/mL, *p* < 0.05), but significantly stronger than that of BHT (IC_50_ value = 226.10 ± 9.00 μg/mL, *p* < 0.05). Then followed by n-BuOH, CE, AQ, PE, and DCM, with the IC_50_ values of (379.11 ± 28.85, 400.25 ± 15.78, 600.30 ± 35.97, 1838.67 ± 57.66, and 3589.61 ± 1.98 μg/mL).

For FRAP, Fe^3+^-TPTZ would be reduced when antioxidants were added in acidic conditions and produced blue-purple Fe^2+^-TPTZ, which could be detected at 593 nm (34). According to Figure 1C, the EtOAc fraction showed the highest FRAP value (889.62 ± 20.44 μg FeSO_4_/mg DE), followed by n-BuOH, DCM, CE, AQ, and PE, with the values of (443.19 ± 3.57, 358.81 ± 5.78, 160.64 ± 0.83, 92.06 ± 3.78, and 24.60 ± 0.26 μg/mL). EtOAc still presented the strongest FRAP, and PE was the weakest. We also found that EtOAc and n-BuOH fractions were weaker than that of Vc but stronger than that of BHT (*p* < 0.05). Thus, taken together, the EtOAc fraction had excellent antioxidant properties compared with all other fractions and commercial antioxidants Vc and BHT, which exhibited a remarkable antioxidant potential.

**FIGURE 2:**
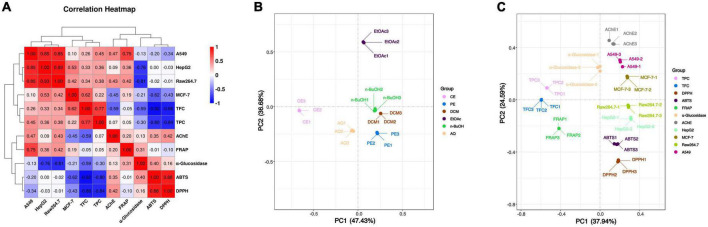
Heatmap graph of correlation analysis and principal co-ordinates analysis of BCF crude extract and its fractions. **(A)** Heatmap graph of correlation analysis, *P*-value (ranging from –1 to 1) and corresponding color (blue to red) represented the magnitude of the spearman correlation coefficient (r) in the heatmap; **(B)** PCoA of BCF extract and its fractions; **(C)** PCoA of active ingredient content and biological activity of BCF extract and its fractions.

To further investigate the association between these active components and indicators, the spearman correlation coefficient (*r*-value) was used for heatmap construction, and the strength of the correlation is indicated by the color from red to blue. From Figure 2A, DPPH and ABTS radical scavenging abilities were highly correlated with TPC and TFC with the r value from –0.80 to –0.92 (*p* < 0.01), and the correlation between FRAP and TPC or TFC was not obvious. These results indicated that DPPH and ABTS radical scavenging abilities were determined principally by the phenolic and flavonoid compounds, while FRAP was the result of the comprehensive action of various components in BCF. This was demonstrated by the principal coordinates analysis (PCoA) in Figure 2C, that FRAP was considerably further away from all other indicators.

### α-glucosidase inhibition ability

**FIGURE 3:**
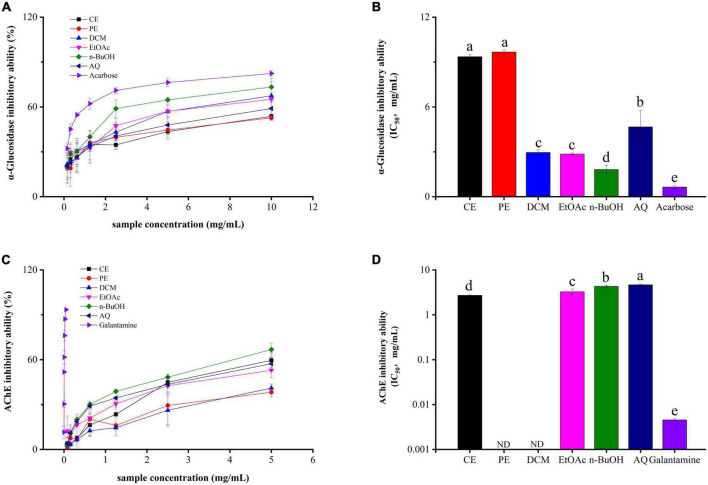
The α-glucosidase **(A,B)** and acetylcholinesterase **(C,D)** inhibitory activities of BCF crude extract and its fractions.

α-Glucosidase inhibitors (AGIs) are first-line drugs to treat diabetes mellitus (DM), and phenolics from plants are excellent sources of AGIs with high efficiency and low side effect (35). From Figures 3A,B, all samples had significantly α-glucosidase inhibitory abilities in a dose-dependent manner. Among these, the n-BuOH fraction gave the strongest α-glucosidase inhibition activity with the IC_50_ value of 1.82 ± 0.29 mg/mL, and its effect is slightly lower than that of the control acarbose (IC_50_ value = 0.64 ± 0.10 mg/mL). Followed by the EtOAc, DCM, AQ, CE, and PE fractions, with IC_50_ value of 2.85 ± 0.076, 2.95 ± 0.18, 4.67 ± 1.09, 9.36 ± 0.13, and 9.67 ± 0.14 mg/mL.

Correlation analyses revealed that flavonoids were the major contributors to the α-glucosidase inhibitory activity (*r* = –0.59, *p* < 0.05), In contrast, lower correlations were found between α-glucosidase inhibitory activity with TPC and antioxidant abilities. This also proved that α-glucosidase inhibitory activity was the combined effect of various bioactive substances in BCF. Previous studies have reported the hypoglycemic activity of BCF *in vitro* and *in vivo* (36, 37), which was in accordance with our experimental results. Given that the *in vivo* hypoglycemic activity of the n-BuOH and EtOAc fractions merit further investigation.

### Acetylcholinesterase inhibition ability

AChE is the common target to treat and prevent Alzheimer’s disease (AD) in the clinic (38). As shown in Figures 3C,D, the CE fraction showed the strongest AChE inhibitory activity with an IC_50_ value of 2.71 ± 0.15 mg/mL, followed by the EtOAc (IC_50_ value = 3.27 ± 0.49 mg/mL), n-BuOH (IC_50_ value = 4.31 ± 0.20 mg/mL), and AQ (IC_50_ value = 4.66 ± 0.15 mg/mL) fractions with IC_50_ values above 3 mg/mL. In addition, PE and DCM fractions failed to attain the IC_50_ value. Taken as a whole, all fractions were significantly weaker than the positive control galantamine (IC_50_ value = 0.0045 ± 0.00030 mg/mL). But the crude extract of BCF and its EtOAc, n-BuOH and AQ fractions still showed inhibitory activity to AChE.

Correlation analyses showed that a higher relationship of AChE inhibitory activity with A549 (*r* = 0.72, *p* < 0.01) and MCF-7 (*r* = 0.61, *p* < 0.05) biological activities (Figure 2), EtOAc fractions of BCF show the better activity. Similarly, the AChE inhibitory activity of the BCF crude extract and its fractions measured by us was similar to that of Sinha et al., and the AChE inhibitory activity of the BCF CE fraction was also significantly lower than that of the positive control group galantamine (39).

### Cell proliferation inhibitory activity

**FIGURE 4:**
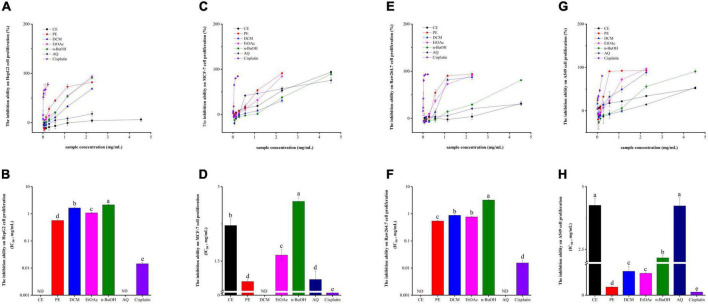
The HepG2 cell **(A,B)**, MCF-7 cell **(C,D)**, Raw264.7 cell **(E,F)**, A549 cell **(G,H)** proliferation inhibitory activity of BCF crude extract and its fractions.

It is well known that oxidative stress is involved in a range of chronic diseases, like cancers, diabetes, cardiovascular diseases, Alzheimer’s disease, and Parkinson’s disease (40). Therefore, in the present investigation, HepG2, MCF-7, Raw264.7, and A549 cells were used to evaluate the antitumor or cell proliferative inhibitory abilities of BCF and its fractions. As described in Figures 4A,B, the PE fraction exhibited the strongest HepG2 cell proliferation inhibition activity with an IC_50_ value of 0.57 ± 0.041 mg/mL. Followed by the EtOAc, DCM, and n-BuOH fractions, with the IC_50_ values of 1.08 ± 0.051, 1.65 ± 0.012, and 2.16 ± 0.068 mg/mL. While the IC_50_ values for CE fraction and AQ fraction could not be calculated. Despite the strong inhibitory effect of PE and other fractions, they showed a considerably weaker effect than the positive control cisplatin (IC_50_ value = 0.014 ± 0.0016 mg/mL).

From Figures 4C,D, the PE fraction still showed the strongest potential in inhibiting MCF-7 proliferation (IC_50_ value = 1.09 ± 0.036 mg/mL), followed by the AQ fraction (IC_50_ value = 1.13 ± 0.17 mg/mL), but not significantly different between them. Then they were the EtOAc fraction (IC_50_ value of 1.62 ± 0.11 mg/mL), CE fraction (IC_50_ value = 2.22 ± 0.013 mg/mL), and n-BuOH fraction (IC_50_ value = 2.70 ± 0.087 mg/mL). Only the inhibition activity of DCM was not detected. In comparison, cisplatin, used as a positive control, had an IC_50_ value of 0.046 ± 0.0028 mg/mL.

Raw264.7 cell is a mouse peritoneal macrophage cell line established from a tour induced by Abelson murine leukemia virus, while macrophages play an important role in inflammation and immune defense responses (41, 42). Thus, we examined the effects of BCF on Raw264.7 cells and the results were presented in Figures 4E,F. Unlike HepG2 and MCF-7, the PE fraction had the highest activity to inhibit Raw264.7 cell proliferation (IC_50_ value = 0.55 ± 0.027 mg/mL), followed by the EtOAc (IC_50_ value = 0.77 ± 0.046 mg/mL), DCM (IC_50_ value = 0.89 ± 0.023 mg/mL), and the n-BuOH (IC_50_ value = 3.22 ± 0.22 mg/mL) fractions, and the CE and AQ fractions still did not provide IC_50_ values, while the IC_50_ value for cisplatin was 0.015 ± 0.0039 mg/mL.

According to Figures 4G,H, similar to Raw264.7 cell proliferation inhibitory ability, the PE fraction exhibited the strongest activity against A549 cell proliferation with an IC_50_ value of 0.33 ± 0.018 mg/mL, which was not significantly different from the positive control group cisplatin (IC_50_ value = 0.13 ± 0.0045 mg/mL). The EtOAc fraction (IC_50_ value = 0.87 ± 0.027 mg/mL) also had stronger ability to inhibit A549 cell, next was the DCM fraction (0.95 ± 0.020 mg/mL), and there were no differences between them. Followed by the n-BuOH fraction (IC_50_ value = 2.16 ± 0.091 mg/mL), AQ fraction (IC_50_ value = 4.24 ± 0.33 mg/mL), and the CE fraction (IC_50_ value = 4.26 ± 0.27 mg/mL), and the CE and AQ fractions still had no significant activity.

Correlation analysis showed that the HepG2 cell proliferation inhibition activity of BCF were significantly associated with Raw264.7 (*r* = 0.93, *p* < 0.01), A549 (*r* = 0.85, *p* < 0.05) and MCF-7 (*r* = 0.53, *p* < 0.05) cell proliferation inhibition abilities, but was no significant associations with TPC, TFC, and the antioxidant activities. A549 cell proliferation inhibition activity was significantly correlated with FRAP (*r* = 0.75, *p* < 0.01) in addition to HepG2 and Raw264.7 (*r* = 0.85, *p* < 0.01). In addition, the MCF-7 cell proliferation inhibition ability was only significantly correlated with HepG2 cell proliferation inhibition ability (*r* = 0.53, *p* < 0.05). Those results were also consistent with the distances between these indicators in the PCoA analysis plot (Figure 2). These combined findings suggested that α-glucosidase and AChE inhibitory activity, and anticancer activity may be due to the combined effects of the complex ingredients in BCF, rather than a single component. Overall, BCF extract and its fractions showed had demonstrated good antitumor or cell proliferative inhibitory abilities, especially its PE, DCM, EtOAc, and n-BuOH fractions. Although the effects of these fractions were weaker than the positive control cisplatin, considering that BCF could be ingested greatly as a food resource, it showed a potential good protective role against cancer.

### High-performance liquid chromatography analysis

**FIGURE 5:**
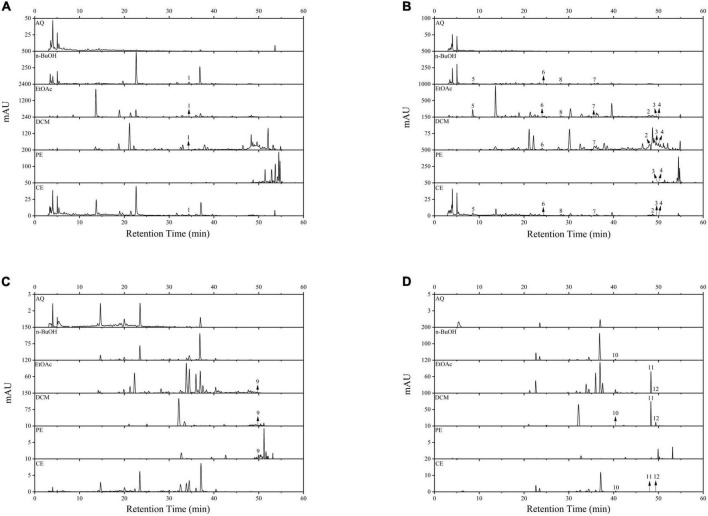
The HPLC spectrum of BCF crude extract and its fractions. **(A)** Wavelength at 256 nm; **(B)** wavelength at 280 nm; **(C)** wavelength at 340 nm; **(D)** wavelength at 360 nm. 1, Rutin; 2, Resveratrol; 3, Baicalein; 4, Hesperetin; 5, Gallic acid; 6, Epicatechin; 7, (–)-Epicatechin gallate; 8, Dihydromyricetin; 9, Apigenin; 10, Myricetin; 11, Quercetin; 12, Luteolin.

In this study, a total of three phenolic acids and nine flavonoids were identified and quantified in BCF and its fractions by using HPLC-DAD, and the results were displayed in Figure 5 and Table 2. HPLC chromatograms confirmed differences among the different fractions, and the EtOAc and CE fractions had the most chromatographic peaks. By comparison of the retention times and peak shapes of detected peaks with those of authentic standards, we identified 11, 11, 10, 6, and 3 compounds in the CE, EtOAc, DCM, n-BuOH, and PE fractions, respectively. But no compounds were detected in the AQ fraction. Among the identified compounds, rutin, gallic acid, epicatechin, (–)-epicatechin gallate, quercetin, and luteolin were the main compounds. In all fractions, the EtOAc fraction exhibited the highest contents of rutin (3.94 ± 0.12 μg/mg E), resveratrol (0.41 ± 0.02 μg/mg E), gallic acid (9.86 ± 1.03 μg/mg E), epicatechin (3.61 ± 0.41 μg/mg E), and dihydromyricetin (0.59 ± 0.08 μg/mg E). These compounds had been reported to possess excellent antioxidants, AGIs, and anticancer activities, which was the possible reason for the strong bioactivity of the EtOAc fraction (30, 36, 43).

**TABLE 2:** The contents of individual phenolic compounds in the BCF crude extract and its fractions.

Compounds	CE	PE	DCM	EtOAc	n-BuOH	AQ
(μg/mg E)						
Rutin	0.21 ± 0.02^c^	ND	0.22 ± 0.04^c^	3.94 ± 0.12^a^	3.46 ± 0.08^b^	ND
Resveratrol	0.33 ± 0.01^b^	ND	0.057 ± 0.001^c^	0.41 ± 0.02^a^	ND	ND
Baicalein	0.71 ± 0.03^b^	0.72 ± 0.03^b^	0.92 ± 0.06^a^	0.72 ± 0.04^b^	ND	ND
Hesperetin	0.18 ± 0.01^b^	0.21 ± 0.05^b^	0.47 ± 0.07^a^	0.21 ± 0.03^b^	ND	ND
Gallic acid	2.88 ± 0.13^b^	ND	ND	9.86 ± 1.03^a^	0.77 ± 0.05^c^	ND
Epicatechin	0.51 ± 0.12^c^	ND	2.96 ± 0.17^b^	3.61 ± 0.41^a^	0.42 ± 0.03^cd^	ND
(–)-Epicatechin gallate	3.59 ± 0.32^a^	ND	3.57 ± 0.22^a^	3.56 ± 0.35^a^	3.57 ± 0.28^a^	ND
Dihydromyricetin	0.33 ± 0.05^b^	ND	ND	0.59 ± 0.08^a^	0.055 ± 0.03^a^	ND
Apigenin	ND	0.23 ± 0.03^c^	1.62 ± 0.43^a^	0.76 ± 0.27^b^	ND	ND
Myricetin	0.81 ± 0.01^b^	ND	0.91 ± 0.02^a^	ND	0.53 ± 0.04^c^	ND
Quercetin	0.25 ± 0.03^c^	ND	4.88 ± 0.17^a^	4.62 ± 0.13^b^	ND	ND
Luteolin	0.27 ± 0.05^c^	ND	3.15 ± 0.31^a^	2.99 ± 0.23^b^	ND	ND

The different letters in the same line represent significant difference (*p* < 0.05).

**FIGURE 6:**
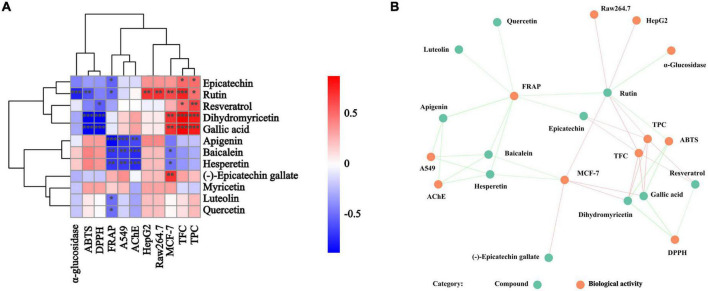
Correlation analysis of individual compounds and biological activities from BCF crude extract and its fractions. *P*-value (ranging from –1 to 1) and corresponding color (blue to red), *p* < 0.05 was considered as significant (*), *p* < 0.01 as highly significant (**), and *p* < 0.001 as very significant (***). **(A)** Correlation heat map; **(B)** correlation network diagram, and each line connects the bioactivity or interphase of the compound *p* < 0.05.

The correlational analysis between these compounds and the biological activities was further conducted. From Figure 6, we found that rutin, gallic acid, epicatechin, quercetin, and luteolin were the main contributors to the biological activities of BCF and its fractions. Of them, rutin, gallic acid, and dihydromyricetin were significantly associated with ABTS radical scavenging ability (*p* < 0.05 or 0.01), while gallic acid, dihydromyricetin, and resveratrol were strictly linked to DPPH radical scavenging ability, but FRAP was tightly linked to epicatechin, rutin, apigenin, baicalein, hesperetin, luteolin, and quercetin. For α-glucosidase activity, its main contributor was rutin. However, apigenin, baicalein, and hesperetin determined not only FRAP but also AChE inhibitory activity and the A549 cell proliferation inhibition ability. In addition, the MCF-7cell proliferation inhibitory ability was mainly influenced by rutin, dihydromyricetin, gallic acid, baicalein, hesperetin, and (–)-Epicatechin gallate. Curiously, Raw264.7 cell proliferation inhibitory ability was only significantly related to rutin. The above indicated that these biological activities of BCF were often affected by multiple compounds, rather than a single ingredient. It was further demonstrated by the correlation network diagram (Figure 6B).

### Ultra-performance liquid chromatography quadrupole-time-flight mass spectrometry analysis

**FIGURE 7:**
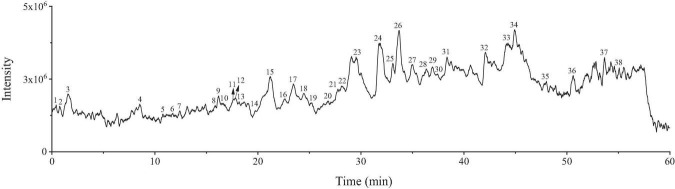
The total ion current (TIC) chromatogram of EtOAc fraction.

Our experiments above demonstrated that the EtOAc fraction of BCF showed the best biological activity in all fractions, therefore, UPLC/Q-TOF-MS was utilized in this study to characterize the chemical composition of the EtOAc fraction. By comparing their mass spectra with the mass spectra available from the PubChem, MassBank database, and other databases, with MS spectra and MS fragmentation patterns published in the literature, a total of 38 compounds were identified, including 24 phenolic acids and 6 flavonoids, and other compounds. The total ion current (TIC) chromatogram of the sample was depicted in Figure 7, whereas the overall identified compounds were reported in Table 3.

**TABLE 3:** The MS information of tentatively identified compounds from EtOAc fraction.

No.	RT (time)	MS [M-H]	Molecular formula	Molecular weight	Concentration (ppm)	MS/MS fragments	Proposed compounds	Reference
1	1.536	191.0563	C_7_H_12_O_6_	191.055	4	127.0403, 111.0105	Quinic acid	(49)
2	1.58	133.0134	C_4_H_6_O_5_	133.0132	2.6	115.0049	Malic acid	(49)
3	4.58	169.0143	C_7_H_6_O_5_	169.0132	6.7	125.0286	Gallic acid	(50)
4	8.334	153.0192	C_7_H_6_O_4_	153.0182	6	109.0314	Protocatechuic acid	(51)
5	11.104	175.0602	C_7_H_12_O_5_	175.0601	2.8	85.0652, 113.0595, 115.0414	Hydroxy-methylglutaric acid	(52)
6	12.216	227.0555	C_10_H_12_O_6_	227.055	2.4	153.0198, 137.0210,108.0190	1-O-(2,4-dihydroxy) benzoylglycerol	(53)
7	13.43	137.0241	C_7_H_6_O_3_	137.0233	6.4	93.0373, 65.0411	Protocatechualdehyde	(49)
8	16.26	167.0347	C_8_H_8_O_4_	167.0339	5.7	108.0242, 123.0493	Vanillic acid	(50)
9	16.511	177.0199	C_9_H_6_O_4_	177.0182	9.6	133.0296, 149.0368	5,7-Dihydroxy coumarin	(54)
10	16.885	353.0865	C_16_H_18_O_9_	353.0867	–0.5	191.0662	Chlorogenic acid	(49)
11	17.72	179.0346	C_9_H_8_O_4_	179.0339	4.2	135.0453	Caffeic acid	(49)
12	18.143	197.0456	C_9_H_10_O_5_	197.0444	5.6	135.0476, 179.0028	Danshensu	(47)
13	18.699	281.1387	C_15_H_22_O_5_	281.1384	1.4	123.0813, 137.1012, 179.1426, 207.1408	Artemisinin	(55)
14	20.475	225.1137	C_12_H_18_O_4_	225.1121	7.1	59.0148, 97.0660, 147.0823, 151.0395	Senkyunolide J/N 1 or Senkyunolide J/N 2	(56)
15	21.119	421.0781	C_19_H_18_O_11_	421.0765	3.7	259.0249, 271.0248, 285.0409, 301.0350	Mangiferin	(57)
16	22.662	209.0453	C_10_H_10_O_5_	209.0444	3.9	76.0341, 120.0241, 165.0591	5-Hydroxyferulic acid methyl ester	(56)
17	23.492	163.0405	C_9_H_8_O_3_	163.039	9.3	119.0537, 93.0365	p-Coumaric acid	(58)
18	24.592	359.0951	C_15_H_20_O_10_	359.0973	–5.9	197.0245, 138.0786, 153.0805, 167.0016	Glucosyringic acid	(59)
19	25.226	385.1114	C_17_H_22_O_10_	385.1129	–4	223.0288, 179.0386	1-O-sinapoyl-β-D-glucose	(49)
20	27.754	435.0922	C_20_H_20_O_11_	535.0922	0	345.0621, 315.0523, 300.0293, 272.0337, 244.0407	7-O-Methylmangiferin	(49)
21	28.143	337.0925	C_16_H_18_O_8_	337.0918	2	163.0415	5-p-Coumaroylquinic acid	(49)
22	28.963	431.0973	C_21_H_20_O_10_	431.0973	0	283.0626, 311.0583	Vitexin	(60)
23	29.602	337.0925	C_16_H_18_O_8_	337.0918	2	163.0415	5-p-Coumaroylquinic acid isomer	(61)
24	31.899	477.0672	C_21_H_18_O_13_	477.0664	1.6	301.0356, 179.0020, 151.0039	Quercetin 3-O-glucuronide	(62)
25	33.188	433.077	C_20_H_18_O_11_	433.0765	1	151.0042, 271.0262, 300.0294, 301.0376	Avicularin	(63)
26	33.745	327.1066	C_15_H_20_O_8_	327.1074	–2.6	282.1140, 206.0465, 165.0146, 121.0630	Monohydroxymethoxyacetophenone hexose	(64)
27	35.043	461.0713	C_21_H_18_O_12_	461.0715	–0.4	285.0404, 257.0455, 229.0515	Kaempferol 3-O-glucuronide	(62)
28	36.452	417.0817	C_20_H_18_O_10_	417.0816	0.3	285.0432, 284.0336, 257.0446, 255.0326, 227.0364	Kaempferol-3-O-pentoside	(58)
29	37.09	343.2021	C_18_H_32_O_6_	343.2115	1.6	325.2035, 229.1446, 201.1143, 185.1178, 171.1048	Dihydroxyoctadecenedioic acid isomer	(55)
30	37.547	433.1128	C_21_H_22_O_10_	433.1129	–0.3	271.0078, 313.0359	Naringenin-C-glucoside	(65)
31	38.811	345.2282	C_18_H_34_O_6_	345.2272	3.1	327.2169, 265.1852, 201.1132, 171.1026, 165.1288	Dihydroxyoctadecanedioic acid	(55)
32	42.166	301.0361	C_15_H_10_O_7_	301.0343	6.2	179.0006, 151.0052	Quercetin	(49)
33	44.108	327.2166	C_18_H_32_O_5_	327.2166	–0.1	229.1465, 211.1363, 171.1030,	9,12,13-Trihydroxy-octadecadienoic acid	(57)
34	44.948	329.2334	C_18_H_34_O_5_	329.2322	3.6	211.1326, 171.1022	Trihydroxy octadecenoic acid	(49)
35	47.874	313.2378	C_18_H_34_O_4_	313.2373	1.5	183.1408	Dihydroxy octadecenoic acid	(49)
36	51.725	277.2167	C_18_H_30_O_2_	277.2162	1.9	233.2383, 197.1319	Linolenic acid	(49)
37	53.613	255.2327	C_16_H_32_O_2_	255.2319	3.3	237.2116	Palmitic acid	(49)
38	55.53	283.2635	C_18_H_36_O_2_	283.2632	1.1	229.1202, 211.1476	Stearic acid	(49)

The result of MS analysis revealed the presence of various impressive active compounds in the EtOAc fraction of BCF, such as gallic acid, quercetin, protocatechuic acid, quinic acid, and artemisinin. Surprisingly, most of these compounds have been proven to possess various biological activities. For example, the quinic acid derivatives with caffeoyl moiety have been reported to show significant DPPH radical scavenging activity (44). Quercetin could ameliorate the oxidative stress-induced apoptosis of seminal vesicle cells by activating Nrf2 (nuclear transcription factor) in type 1 diabetic rats (45). Artemisinin also could protect against cerebral ischemia and reperfusion injury *via* inhibiting the NF-kB (nuclear factor kappa B) pathway (46). Moreover, the epicatechin derivative had anticancer, antioxidant, hepatoprotective, anti-inflammatory, and anti-microbial properties (47). The aforementioned results indicate that these compounds possibly are the material bases of the EtOAc fraction of BCF, that play a key pharmacological role.

### Network pharmacology analysis

Network pharmacology is a new subject based on the theory of systems biology, which conducts network analysis on “drugs, diseases, genes, and targets,” and explores the internal connection between the multiple targets of drug treatment of diseases (31). Based on computer analysis technology, by constructing a “component-target-pathway” network, the interaction relationship between drug components and diseases can be systematically analyzed as a whole, and the potential role of active ingredients in traditional Chinese medicine in treating diseases can be revealed from the perspective of molecular biology (32). It is of great significance to the research on the mechanism of drug action and the innovative research and development of new Chinese medicines.

**FIGURE 8:**
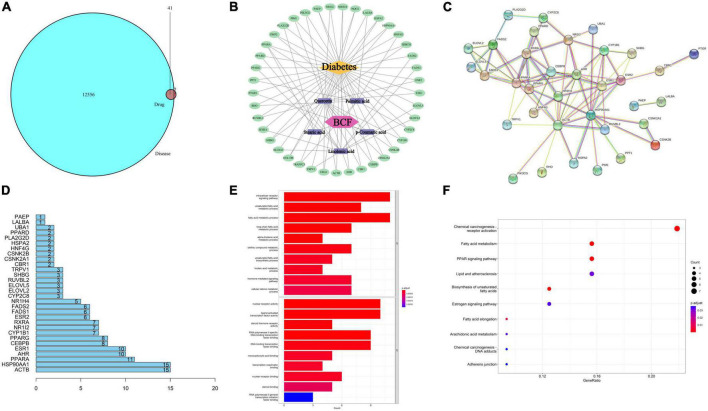
Enriched KEGG pathways based on compounds identified by mass spectrometry in the EtOAc fraction of BCF. **(A)** The relationship between drug targets and disease targets; **(B)** BCF active ingredient-anti-diabetic target network; **(C)** PPI network of potential antidiabetic targets of BCF; **(D)** core gene histogram in PPI; **(E)** column chart of GO functional enrichment results of BCF potential anti-diabetic targets; **(F)** KEGG signaling pathway enrichment analysis bubble plot.

According to the previous results of mass spectrometry analysis, we retrieved the target of these 38 compounds in the pharmacology database and analysis platform (TCMSP) and performed gene ID conversion for the target of the component in the protein database (Uniprot). Then, all the disease targets related to diabetes were searched in the Human Gene Database (GeneCards) and the Comparative Toxic Gene Database (CTD), and the disease targets with strong correlation were screened and then merged and deduplicated, and the action targets of drug components were compared with diabetes. After the intersection of related disease targets, 41 common targets were obtained (Figure 8A). Using Cytoscape 3. 7. 2 software, a visual network diagram of BCF-active ingredient-target-disease was established (Figure 8B). We found that p-coumaric acid, quercetin, linolenic acid, palmitic acid, and stearic acid were the main antidiabetic compounds in BCF. The STRING online platform was used to analyze the PPI protein interaction network of 41 potential anti-diabetic targets of BCF, and the PPI network diagram was obtained (Figure 8C). The 41 target genes were drawn as a histogram in the PPI, and the top 30 targets with the highest degree values were drawn as a core gene histogram, as shown in Figure 8D. The top 10 disease targets involved include ACTB, HSP90AA1, PPARA, AHR, ESR1, CEBPB, PPARG, CYP1B1, NR1I2, and RXRA. Gene ontology (GO) function enrichment analysis was performed on the 41 core targets of BCF acting on type diabetes, and the results were shown in Figure 8E. In terms of biological process (BP), it mainly involved intracellular receptor signaling pathways, unsaturated fatty acid metabolism, fatty acid metabolism, alkene compound metabolism, and cellular ketone metabolism. In terms of molecular function (MF), it mainly involved DNA transcription factor binding, enzyme binding, receptor binding, etc. This indicated that BCF could regulate a variety of biological responses involved in the treatment of diabetes. The KEGG pathway analysis was carried out on the 41 core targets of BCF using DAVID and Hiplot databases, and they were sorted according to the number of genes contained in the pathway. The top 10 significant pathway information were listed in Figure 8F, which included chemical oncogenic receptor activation, fatty acid metabolism, PPAR signaling, lipids and atherosclerosis, unsaturated fatty acid biosynthesis, estrogen signaling, and peanut Tetraenoic acid metabolism, etc. In combination, these results demonstrate that the treatment of diabetes by using BCF is a relatively complex mechanism, which needs further study.

## Discussion

In this study, we have carried out a comprehensive evaluation of the antioxidant activities, α-glucosidase and AChE inhibitory activities, antitumor activities, and phytochemical constituents of BCF and its different polar fractions.

The results showed that BCF contained abundant active components and possessed excellent biological activities, especially its EtOAc fraction. Besides having the highest TPC and TFC, the EtOAc fraction also exhibited the strongest DPPH and ABTS free scavenging activity, and FRAP. Its antioxidant activities were comparable and even higher than the positive controls Vc or BHT. These results suggested that BCF, especially its EtOAc fraction, could potentially be developed as a natural antioxidant.

In addition to the EtOAc fraction, the other fractions of BCF also displayed good biological activities in other indicators. For instance, the n-BuOH fraction had not less effective than the EtOAc fraction in inhibition of α-glucosidase. The PE fraction even showed stronger anticancer activity than the EtOAc fraction. Considering that Traditional Chinese medicine is characterized by multiple components and multiple targets, a follow-up study focusing on the different properties of different polar fractions of BCF is needed. These results also suggest that multiple solvents with different polarities rather than a single solvent might be more suitable for the extraction of these compounds in medicinal or edible plants, and EtOAc is conducive to the enrichment of phenolic acids and flavonoids, which is due to its moderate polarity. The above results are consistent with our previous research results (48).

By HPLC analysis, we identified and quantified the main active compounds of BCF and its fractions, which were already reported to be found in BCF. Although the results cannot provide a complete picture of the difference in the species and content of all compounds between each fraction, they still revealed the differences in the major compounds between these fractions, and also strongly supported the result of differences in the biological activity of these fractions. We further revealed the correlation and interaction between active compounds and biological activities and speculated the key active compounds for the pharmacologic effects of BCF, which might provide a foundation for further development of targeted drugs. Previous studies on BCF were not comprehensive and in-depth, our study effectively compensated for these deficiencies, and could provide more useful information on the key compounds of BCF. In addition, a similar research method was used by Miklavcic et al. to research tomatoes, which suggested this approach can be widely applicable to many plants (30).

Considering that the EtOAc fraction showed the best overall performance in whole biological activities, its phytochemical profiles were characterized using MS analysis, from which many compounds which had been shown to have a variety of beneficial and biological activities were identified. This not only supported the HPLC results but also explained the reason for the good comprehensive properties of the EtOAc fraction.

Based on this, we used network pharmacology analysis to predict the pharmacological mechanism by which BCF affects diabetes. The results indicated that BCF can exert its therapeutic effects by affecting multiple pathways and multiple targets along each pathway. Although the most likely targets and pathways of BCF effect on diabetes were obtained, such as chemical oncogenic receptor activation, fatty acid metabolism, PPAR signaling, etc, this hypothesis still requires further experimental verification. Unlike other network pharmacology research was only based on the public compounds database in the network, our research was based on our mass spectrometry analysis results, which could more truly reflect the compounds and their action mechanisms in BCF.

## Conclusion

In summary, BCF exhibited excellent antioxidant activity, α-glucosidase and anticancer activity, especially its EtOAc fraction, and could be applied as potential resources for treating diabetes mellitus and cancer. This work could provide the theoretical basis for the development of BCF. However, the biological activity of BCF and its fractions in this study is only limited to *in vitro* research, and further verification through animal experiments is required.

## Data availability statement

The original contributions presented in this study are included in the article/supplementary material, further inquiries can be directed to the corresponding author/s.

## Author contributions

FW and XX: methodology. ZW, PX, and KY: validation and writing—review and editing. XX: formal analysis. JY: data curation. KY: writing—original draft preparation. JS: supervision. XZ and XH: project administration. JS and ZW: funding acquisition. All authors have read and agreed to the published version of the manuscript.

## Footnotes

1
https://old.tcmsp-e.com/tcmsp.php

2
www.ncbi.nlm.nih.gov/pccompound/

3
http://sea.bkslab.org/

4
https://www.genecards.org/

5
https://string-db.org/

6
https://david.ncifcrf.gov/summary.jsp

7
www.omicshare.com
